# A long noncoding RNA binding to QKI-5 regulates germ cell apoptosis via p38 MAPK signaling pathway

**DOI:** 10.1038/s41419-019-1941-2

**Published:** 2019-09-20

**Authors:** Kai Li, Shunshun Zhong, Yanyun Luo, Dingfeng Zou, Mengzhen Li, Yahui Li, Yan Lu, Shiying Miao, Linfang Wang, Wei Song

**Affiliations:** Department of Biochemistry and Molecular Biology, State Key Laboratory of Medical Molecular Biology, Institute of Basic Medical Sciences, Chinese Academy of Medical Sciences and Peking Union Medical College, Beijing, 100005 China

**Keywords:** Spermatogenesis, Long non-coding RNAs

## Abstract

Spermatogenesis is the complex process of male germline development and requires coordinated interactions by multiple gene products that undergo strict developmental regulations. Increasing evidence has suggested that a number of long noncoding RNAs (lncRNAs) may function as important regulatory molecules in various physiological and pathological processes by binding to specific proteins. Here, we identified a subset of QKI-5-binding lncRNAs in the mouse testis through the integrated analyses of RNA immunoprecipitation (RIP)-microarray and biological verification. Among the lncRNAs, we revealed that NONMMUT074098.2 (*Lnc10*), which was highly expressed in the spermatogonia and spermatocytes of the testis, interacted with QKI-5. Furthermore, *Lnc10* depletion promoted germ cell apoptosis via the activation of p38 MAPK, whereas the simultaneous knockdown of QKI-5 could rescue the apoptotic phenotype and the activation of p38 MAPK, which were induced by the loss of *Lnc10*. These data indicated that the *Lnc10*-QKI-5 interaction was associated with the regulatory roles of QKI-5 and that the *Lnc10*-QKI-5 interaction inhibited the regulation of QKI-5 on the downstream p38 MAPK signaling pathway. Additionally, we functionally characterized the biological roles of *Lnc10* and found that the knockdown of *Lnc10* promoted the apoptosis of spermatogenic cells in vivo; this suggested that *Lnc10* had an important biological role in mouse spermatogenesis. Thus, our study provides a potential strategy to investigate the biological significance of lncRNA-RBP interactions during male germline development.

## Introduction

Spermatogenesis refers to the complicated, yet highly ordered, process of continuous production of haploid spermatozoa from diploid spermatogonia, which proceeds through mitosis, meiosis, and spermiogenesis inside the testes^1,2^. This series of processes involves the coordinated interactions of multiple gene products that undergo strict developmental regulations in time and space^3^. Thus, identifying key regulatory gene products will potentially be useful in elucidating the molecular mechanism of spermatogenesis.

Long noncoding RNAs (lncRNAs) are defined as transcripts that are longer than 200 nucleotides (nt) and have little protein-coding potential^4^. Accumulating evidence shows that lncRNAs are emerging as important regulatory gene products in various physiological and pathological processes^5–7^. Over the past years, genome-wide transcriptome analyses of lncRNA repertoires have demonstrated that lncRNAs are highly organ-specific and preferentially expressed in testes^8^. Furthermore, recent studies have demonstrated the dynamic expression patterns during male germline development, which suggests potential functional roles for lncRNAs in spermatogenesis^9–12^. A testis-specific lncRNA, *Tslrn1*, is highly expressed during the pachytene stage, and *Tslrn1* deletion does not affect normal male fertility but causes a significant reduction in spermatozoa number^13^. Additionally, *lncRNA033862* controls spermatogonial stem cell (SSC) self-renewal and survival by regulating the expression of GDNF receptor alpha1 (*Gfrα1*)^14^. Despite these promising findings, most of the lncRNAs systematically identified in the testis have not been functionally characterized in vivo in a mouse model. One emerging theme is that lncRNAs may exert their functional role by interacting with specific target proteins and act as decoys, guides, or scaffolds^15,16^. Recently, an integrated analysis of large-scale high-throughput sequencing of immunoprecipitated RNAs after cross-linking (CLIP-Seq) and RNA-Seq datasets respectively identified 21,073 and 1662 lncRNA-RNA binding protein (RBP) interactions in humans and mice^17^. A schizophrenia-associated lncRNA, Gomafu, has been found to be involved in the alternative splicing of schizophrenia pathology-related genes through its direct interaction with Quaking (QKI) and serine/arginine-rich splicing factor 1 (SRSF1)^18^. Additionally, lnc-Lsm3b can compete with viral RNA in the binding of retinoic acid-inducible gene-I (RIG-I) monomers, which restricts the conformational shift of the RIG-I protein and prevents downstream signaling in the innate immune response^19^. These findings indicate that the function of many lncRNAs depends on the interaction with functional proteins. However, the biological significance of the interaction of lncRNA with RBP in spermatogenesis remains to be further investigated.

QKI belongs to the signal transduction and activation of RNA (STAR) family of the KH domain-containing RBP^20^. The *Qki* gene produces three major isoforms designated as QKI-5, QKI-6, and QKI-7, respectively^21,22^. QKI has been found to be involved in the regulation of precursor mRNA (pre-mRNA) splicing, mRNA stability, microRNA (miRNA) biogenesis, and circular RNA (circRNA) formation by selectively binding to QKI response element (QRE) located in the target RNAs^23–25^. It is well established that QKI is required for neural development and myelination by regulating oligodendrocyte and Schwann cell differentiation^26^. Since the ubiquitous expression profile of QKI, the interactions of QKI with multiple RNA species have been implicated in various physiological and pathological processes outside the nervous system. QKI is a critical regulator of the vascular smooth muscle cell (VSMC) phenotype by binding to *Myocd* pre-mRNA and regulating alternative splicing^27^. Recently, it has been reported that QKI binds upstream and downstream of circRNA-forming exons to promote circRNA formation during the epithelial-to-mesenchymal transition (EMT)^28^. Additionally, our previous study demonstrates the pivotal role of QKI-5 in regulating primary miR-124-1 processing via a distal RNA motif during erythropoiesis^29^. Despite the functional diversity of specific interactions of QKI with many RNA species, it is largely unknown if QKI can bind to lncRNAs and its functional importance during spermatogenesis.

In this study, we identified 5922 lncRNAs binding to QKI-5 in the mouse testis using an RNA immunoprecipitation (RIP)-microarray analysis. Among them, *Lnc10* exhibited a similar high expression pattern with QKI-5 in the testis. Furthermore, we unveiled that the binding of *Lnc10* to QKI-5 suppressed germ cell apoptosis via inhibiting the activation of the p38 MAPK signaling pathway. Additionally, using a shRNA-mediated functional approach of lncRNA in vivo, we demonstrated that *Lnc10* played a significant regulatory role in mouse spermatogenesis.

## Results

### QKI-5 is highly expressed in the mouse testis

**Fig. 1:**
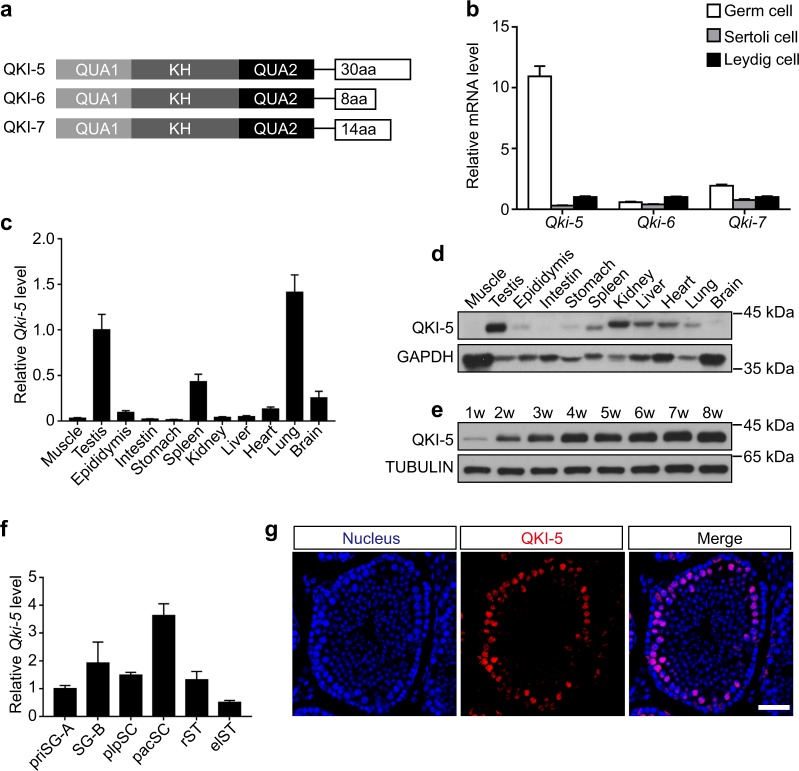
QKI-5 is highly expressed in the mouse testis. **a** Diagram of three major isoforms of the *Qki* gene, QKI-5, QKI-6, and QKI-7. **b** Relative expression levels of *Qki-5*, *Qki-6*, and *Qki-7* (from the cDNA of Germ cells, Sertoli cells and Leydig cells) as detected by qRT-PCR. The data represent the mean ± SEM for three biological replicates that were normalized to the endogenous *Actb* control. **c** qRT-PCR analysis of the *Qki-5* mRNA expression in adult normal mouse tissues. The data represent the mean ± SEM for three biological replicates that were normalized to the endogenous *Gapdh* control. **d** Western blotting analysis of the QKI-5 protein expression in adult normal mouse tissues. GAPDH served as the loading control. **e** Western blotting analysis of the QKI-5 protein expression in normal postnatal mouse testes at 1–8 weeks. TUBULIN served as the loading control; w, week. **f** qRT-PCR analysis of the *Qki-5* mRNA expression in the following 6 distinct germ cells: priSG-A, primitive type A spermatogonia; SG-B, type B spermatogonia; plpSC, preleptotene spermatocyte; pacSC, pachytene spermatocyte; rST, round spermatid; elST, elongating spermatid. The data represent the mean ± SEM for three biological replicates that were normalized to the endogenous *Actb* control. **g** Immunostaining of QKI-5 (red) in adult normal mouse testis. The nuclei were stained with DAPI (blue). Scale bar, 50 μm

The three major isoforms of *Qki* gene, QKI-5, QKI-6, and QKI-7, are differed by ~30 amino acids in their C-termini (Fig. 1a). To detect the expression pattern of the three isoforms, we performed quantitative real-time PCR (qRT-PCR) with cDNA derived from Germ cells, Sertoli cells and Leydig cells^30^. Notably, we observed that QKI-5 was the most abundant isoform in Germ cells (Fig. 1b). However, there was no significant difference among these three isoforms in the Sertoli cells or Leydig cells. Next, we employed qRT-PCR and Western blotting to measure the tissue specificity of QKI-5. The results showed that QKI-5 was most highly expressed in the mouse testis (Fig. 1c, d). Moreover, the expression level of QKI-5 in the mouse testis gradually increased within 4 weeks after birth, and the high expression levels were maintained in adulthood (Fig. 1e). To further assess the expression pattern of QKI-5 during mouse spermatogenesis, we isolated 6 distinct germ cell types from the testes based on the STA-PUT method^31^. A qRT-PCR analysis suggested that the expression level of *QKI-5* was comparable in early spermatogenesis (from priSG-A to plpSC) but was significantly increased in pacSC and gradually reduced in the rST and the elST (Fig. 1f). Furthermore, immunostaining of QKI-5 revealed a major enrichment in the nuclei of spermatocytes (Fig. 1g). Collectively, these data demonstrate that QKI-5 is the major isoform and is highly expressed in the mouse testis.

### Knockdown of QKI-5 inhibits apoptosis in GC1-spg cells via inhibiting the p38 MAPK signaling pathway

**Fig. 2:**
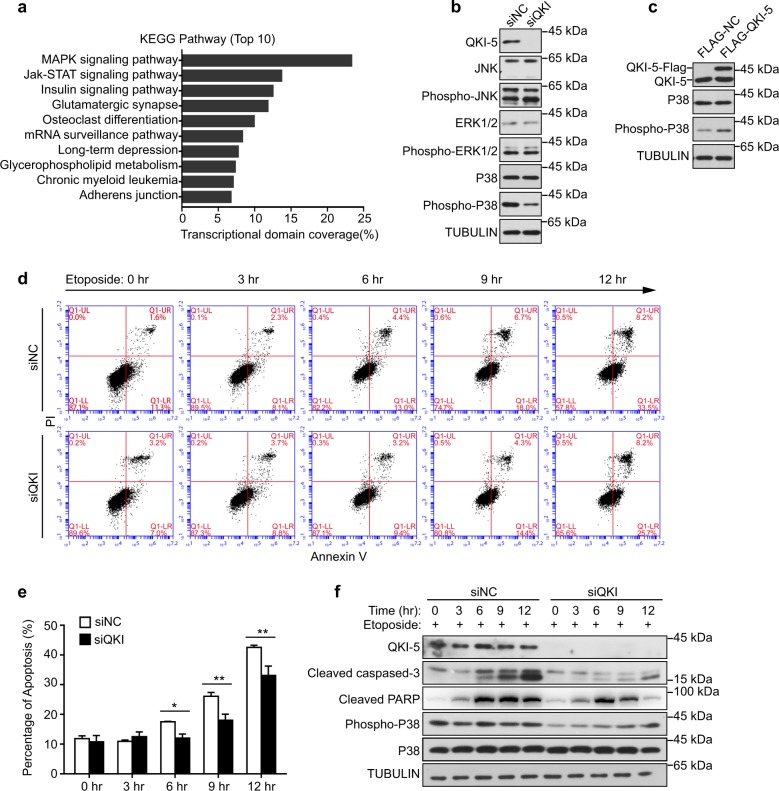
Knockdown of QKI-5 inhibits apoptosis in GC1-spg cells via inhibiting the p38 MAPK signaling pathway. **a** KEGG pathway enrichment analysis of the QKI-5-binding mRNAs as determined by an RIP-microarray in adult mouse testis. **b** Western blotting analysis of the phosphorylation and total levels of ERK1/2, JNK, and p38 MAPK in siNC and siQKI GC1-spg cells. TUBULIN served as the loading control. **c** Western blotting analysis of the phosphorylation and total levels of p38 MAPK in the normal control and QKI-5 overexpression GC1-spg cells. TUBULIN served as the loading control. **d** Representative flow cytometry plots for the apoptosis assays in siNC and siQKI GC1-spg cells following treatment with etoposide (100 μM) for the indicated time. **e** Statistical plots of the percentage of apoptotic cells as determined by flow cytometry in siNC and siQKI GC1-spg cells following treatment with etoposide (100 μM) for the indicated time. The data represent the mean ± SEM of three biological replicates. (*) *p* < 0.05, (**) *p* < 0.01, *t*-test. **f** Western blotting analysis of apoptosis-related proteins, cleaved caspase-3 and cleaved PARP, and p38 MAPK in siNC and siQKI GC1-spg cells following treatment with etoposide (100 μM) for the indicated time. TUBULIN served as the loading control

Given that QKI-5 was the major isoform in the testis and a well-known RNA binding protein, we performed RIP assay to functionally characterize QKI-5 in mouse spermatogenesis. The RNA recovered from RIP was subjected to an Agilent mouse lncRNA microarray to detect the mRNAs and lncRNAs that bind to QKI-5 in the testis. We initially identified 4088 mRNAs according to microarray analysis (Supplemental Table S3). Afterward, Kyoto encyclopedia of genes and genomes (KEGG) pathway analysis and Gene Ontology (GO) term analysis were applied to determine the roles of those mRNAs in spermatogenesis. Notably, we observed that those mRNAs were significantly enriched for the “MAPK signaling pathway” and for the GO term “protein phosphorylation” (Fig. 2a; Supplementary Fig. S1a). The conventional MAPK family mainly comprises ERK1/2, JNK, and p38, each of which is involved in a major signaling pathway. To investigate the major pathway that is modulated by QKI-5, we employed an siRNA-mediated knockdown of QKI-5 in GC1-spg cells and detected the total protein and the phosphorylation levels of ERK1/2, JNK, and p38 MAPK by Western blotting. The results revealed that QKI-5 was knocked down by up to ~90% by siRNA-mediated silencing. Compared with those in the control, the phosphorylation of p38 exhibited a significant decrease, whereas the phosphorylation of ERK1/2 and JNK were largely unaltered following QKI-5 knockdown (Fig. 2b, Supplementary Fig. S1b). In contrast, the overexpression of QKI5-Flag led to increased levels of p38 phosphorylation (Fig. 2c, Supplementary Fig. S1c). These results indicated that QKI-5 was mainly involved in the p38 MAPK signaling pathway.

Previous reports have suggested a role for p38 MAPK in the regulation of apoptosis. Given this model, we examined the effect of QKI-5 knockdown on apoptosis in GC1-spg cells after treatment with etoposide using fluorescence-activated cell sorter (FACS) analysis. Notably, we found that the percentages of total apoptotic cells with QKI-5 knockdown (siQKI) and the normal control (siNC) cells both increased in response to treatment with etoposide in a time-dependent fashion, whereas the knockdown of QKI-5 led to a significant decrease of ~5–9% during 6–12 h compared with the normal control (Fig. 2d, e). Furthermore, Western blotting showed that QKI-5 knockdown diminished the expression level of cleaved caspase-3, cleaved PARP and p38 phosphorylation for the specified periods of time (Fig. 2f). Taken together, these observations suggest that the knockdown of QKI-5 inhibits cell apoptosis via inhibiting the p38 MAPK signaling pathway.

### Identification of lncRNAs binding to QKI-5 in the mouse testis

**Fig. 3:**
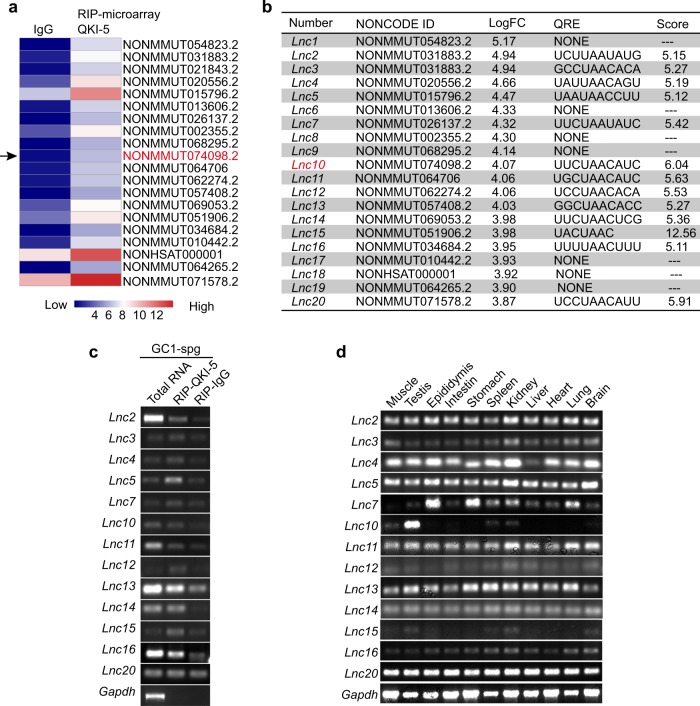
Identification of lncRNAs that bind to QKI-5 in normal adult mouse testis. **a** Heat map of the top 20 lncRNA candidates that bind to QKI-5 as determined by an RIP-microarray. *Lnc10* is highlighted with a black arrow. **b** Preliminary QRE screening of the top 20 lncRNA candidates by RBPDB. Scan threshold, 0.8. **c** RIP-PCR analysis of the 13 lncRNA candidates, including QRE, that bind to QKI-5 in GC1-spg cells. *Gapdh* mRNA served as the negative control and does not contain a QRE. **d** RT-PCR analysis of the 13 lncRNA candidates expression profiles in the normal adult mouse tissues. *Gapdh* mRNA was an endogenous control

We further investigated whether lncRNAs could bind to QKI-5 in the mouse testis. Notably, our microarray analysis identified 5922 lncRNAs that bind to QKI-5 (Supplemental Table S4). The lncRNAs binding to QKI-5 were ranked and shown in Fig. 3a (including the top 20 lncRNAs in the NONCODE database). QKI-5 has been reported to interact with target RNAs via a specific QRE. Considering this notion, we reasoned that the lncRNAs that bind to QKI-5 may contain the QREs as well. We screened the QREs of the top 20 lncRNA candidates by the database of RNA-binding protein specificities (RBPDB, http://rbpdb.ccbr.utoronto.ca). A preliminary QRE screening indicated that 13 lncRNA candidates (65%) contained one or more of the QREs (Fig. 3b). RIP-PCR was performed to further confirm the physical interactions between 13 lncRNA candidates and QKI-5 (Fig. 3c, Supplemental Fig. S2a).

To systematically prioritize the 13 lncRNA candidates for a functional follow-up, we employed RT-PCR to detect the expression profiles of the 13 lncRNA candidates. Of note, we observed that the lncRNA NONMMUT074098.2 (original ID in NONCODE v5.0), named *Lnc10* (mentioned below), was mainly expressed in the mouse testis and exhibited a similar high expression pattern with QKI-5 (Fig. 3d). Accumulating evidence suggests that lncRNAs are more likely biologically significant if they are expressed in a tissue-specific pattern. Given this notion, we reasoned that *Lnc10* binding to QKI-5 may play a potential significant role during mouse spermatogenesis. Collectively, these results demonstrate that lncRNAs, including QRE, could interact with QKI-5 in the mouse testis, and *Lnc10* binding to QKI-5 emerged as the most promising lncRNA candidate for a subsequent functional investigation of mouse spermatogenesis.

### Depletion of *Lnc10* promotes apoptosis in GC1-spg cells via activating the p38 MAPK signaling pathway

**Fig. 4:**
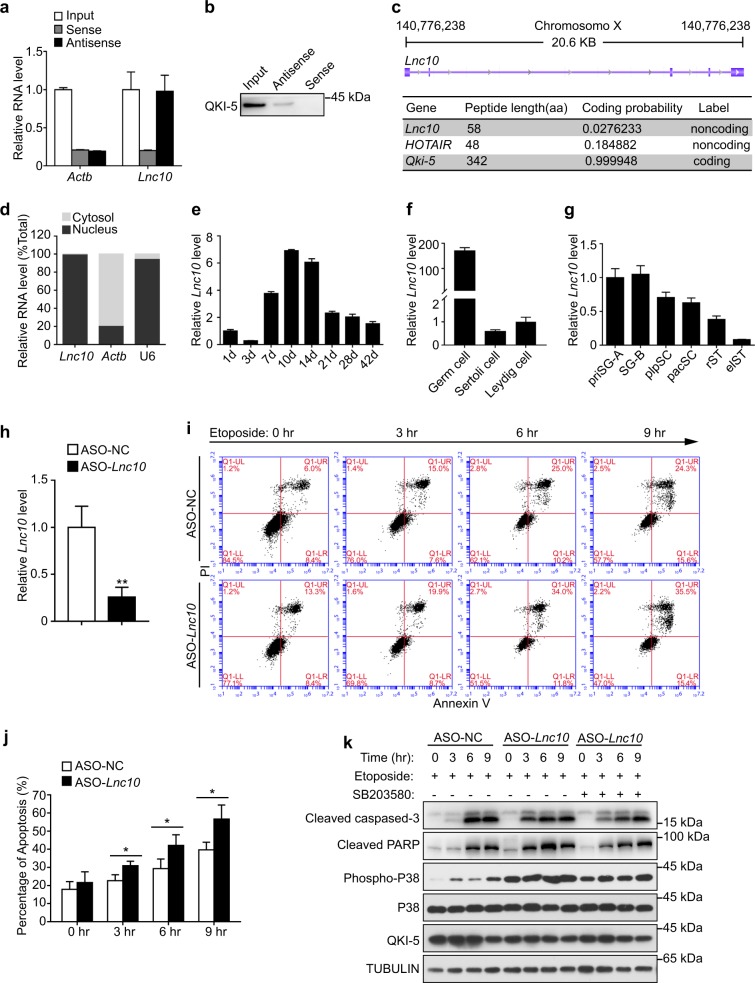
Depletion of *Lnc10* promotes apoptosis in GC1-spg cells via activating the p38 MAPK signaling pathway. **a** qRT-PCR analysis of the enrichment efficiency of a biotinylated antisense probe specifically targeted to *Lnc10*. *Actb* mRNA served as the negative control. The data represent the mean ± SEM. **b** RNA pull-down analysis of the binding of *Lnc10* to QKI-5 in GC1-spg cells as detected by a Western blotting assay. **c** Schematic annotation of the *Lnc10* genomic locus on chromosome X via the UCSC Genome Browser. The blue rectangles represent the exons. The coding potential of *Lnc10* was predicted by the Coding Potential Calculator (CPC2.0) program. The lncRNA HOTAIR was used as a noncoding RNA control. QKI-5 mRNA was used as a coding RNA control. **d** qRT-PCR analysis of the subcellular localization of *Lnc10* by the fractionation of GC1-spg cells. U6 RNA served as a positive control for nuclear gene expression. *Actb* mRNA served as a positive control for cytoplasmic gene expression. **e** qRT-PCR analysis of the *Lnc10* expression in normal postnatal mouse testis for the indicated time. The data represent the mean ± SEM. **f** qRT-PCR analysis of the *Lnc10* expression in cDNA from germ cells, Sertoli cells and Leydig cells. The data represent the mean ± SEM. **g** qRT-PCR analysis of the *Lnc10* expression in 6 distinct germ cells. The data represent the mean ± SEM. **h** qRT-PCR analysis of *Lnc10* knockdown mediated by ASO targeted to *Lnc10*. The data represent the mean ± SEM. **i** Representative flow cytometry plots of apoptosis assays in ASO-NC and ASO-*Lnc10* GC1-spg cells treated with etoposide (100 μM) for the indicated time. **j** Statistical plots of the percentage of apoptotic cells determined by flow cytometry in ASO-NC and ASO-*Lnc10* GC1-spg cells treated with etoposide (100 μM) for the indicated time. The data represent the mean ± SEM. (*) *p* < 0.05, *t*-test. **k** Western blotting analysis of apoptosis-related proteins, cleaved caspase-3 and cleaved PARP, and p38 MAPK in ASO-NC and ASO-*Lnc10* GC1-spg cells treated with etoposide (100 μM) or SB203580 (10 μM) for the indicated time. TUBULIN served as the loading control

To further confirm the interaction between QKI-5 and *Lnc10*, we performed RNA pull-down assay in GC1-spg cells by antisense probes that were designed against endogenous *Lnc10*. A qRT-PCR analysis of the RNA species captured from the RNA pull-down assay revealed a significant enrichment of *Lnc10* (Fig. 4a). The Western blotting analysis showed that QKI-5 proteins could be pulled down by the antisense probes but not by the sense probes (Fig. 4b). Additionally, we detected the interaction between QKI-5 and *Lnc10* from purified fractions of germ cells (SG-B and pacSC) as well (Supplemental Fig. S3a, b). *Lnc10* resides on chromosome X in mice and is composed of five exons; it spans nearly 20.6 kilobases (kb) according to an analysis with the UCSC Genome Browser (Fig. 4c). The Coding Potential Calculator 2.0 (CPC 2.0) computational algorithm predicted that *Lnc10* has a low coding potential and was labeled as a noncoding RNA, similar to HOTAIR, which is a well-defined lncRNA (Fig. 4c). We then examined the subcellular localization of *Lnc10* by cell fractionation followed by qRT-PCR. The results suggest that *Lnc10* is mainly localized in the nucleus, and this is consistent with the localization of the QKI-5 protein (Fig. 4d; Supplemental Fig. S2b).

To assess the expression pattern of *Lnc10* during mouse spermatogenesis, we initially performed qRT-PCR with cDNA derived from postnatal mouse testes. The results revealed that *Lnc10* was stage-specific; a higher transcript level was detected in postnatal day 10 (P10) mouse testes, whereas both newborn and adult mouse testes expressed low levels (Fig. 4e). We then investigated the cell type specificity of *Lnc10* in Germ cells, Sertoli cells and Leydig cells. A qRT-PCR analysis suggested that *Lnc10* was mainly expressed in germ cells, a similar expression pattern to that of QKI-5 (Fig. 4f). We further detected the expression dynamics of *Lnc10* in distinct germ cell and the results showed that the expression of *Lnc10* was comparable in priSG-A and SG-B. Then, a gradual decline was observed in plpSC and pacSC, and the level dramatically reduced in rST and elST (Fig. 4g). These results indicate that *Lnc10* is stage-specific in mouse spermatogenesis and that *Lnc10* is predominantly expressed in spermatogonia and spermatocytes.

Taking into account the regulatory function of QKI-5 on the p38 MAPK signaling pathway and apoptosis, we further determined whether *Lnc10* binding to QKI-5 was likewise involved in the p38 MAPK signaling pathway and apoptosis. We knocked down *Lnc10* via the LncRNA Smart Silencer composed of 3 antisense oligonucleotides (ASO) in GC1-spg cells and examined the effect of knockdown of *Lnc10* on the regulation of the p38 MAPK and apoptosis. Notably, we observed a dramatic ~70% reduction in the expression of *Lnc10* by ASO-mediated silencing (Fig. 4h). In addition, the percentage of total apoptotic cells with *Lnc10* knockdown (ASO-*Lnc10*) and the normal control (ASO-NC) both increased after treatment with etoposide in a time-dependent fashion, while the knockdown of *Lnc10* caused a substantial increase of ~8.3–17% over 3–9 h compared with the normal control (Fig. 4i, j). Furthermore, Western blotting showed that the *Lnc10* knockdown activated the p38 MAPK and promoted the expression of cleaved caspase-3 and cleaved PARP after treatment with etoposide (Fig. 4k). To prove the specificity of p38 MAPK activation in the induction of apoptosis in this system, we treated ASO-*Lnc10* cells with SB203580, a p38 MAPK inhibitor, and then detected cleaved caspase-3, cleaved PARP and the phosphorylation levels of p38 by Western blotting. We observed that such treatment could at least in part revert the effect of ASO-*Lnc10* (Fig. 4k). These data demonstrate that the knockdown of *Lnc10* promotes apoptosis in GC1-spg cells via activating the p38 MAPK signaling pathway.

### *Lnc10* inhibits the QKI-5 downstream p38 MAPK signaling pathway by binding to QKI-5 in GC1-spg cells

**Fig. 5:**
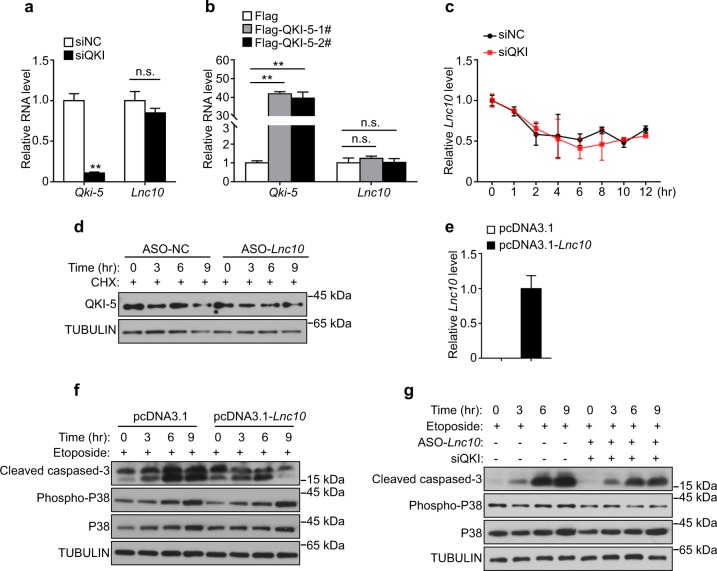
*Lnc10* inhibits the QKI-5 downstream p38 MAPK signaling pathway by binding to QKI-5 in GC1-spg cells. **a** qRT-PCR analysis of the *Lnc10* expression change after QKI-5 knockdown in GC1-spg cells. The data represent the mean ± SEM. (**) *p* < 0.01, (n.s.) *p* > 0.05, *t*-test. **b** qRT-PCR analysis of the *Lnc10* expression change after QKI-5 overexpression in GC1-spg cells. The data represent the mean ± SEM. (**) *p* < 0.01, (n.s.) *p* > 0.05, *t*-test. **c** qRT-PCR analysis of the stability of the *Lnc10* transcript after QKI-5 knockdown followed by treatment with actinomycin D (5 μg/mL) in GC1-spg cells for the indicated time. The data represent the mean ± SEM. **d** Western blotting analysis of QKI-5 expression after *Lnc10* knockdown followed by treatment with CHX in GC1-spg cells for the indicated time. TUBULIN served as a loading control. **e** qRT-PCR analysis of the *Lnc10* overexpression level in GC1-spg cells. The data represent the mean ± SEM. **f** Western blotting analysis of apoptosis-related proteins, cleaved caspase-3, and p38 MAPK in the normal control and *Lnc10* overexpression GC1-spg cells followed by treatment with etoposide (100 μM) for the indicated time. TUBULIN served as a loading control. **g** Rescue assay for the apoptotic phenotype and the activation of p38 MAPK induced by the loss of *Lnc10* after QKI-5 knockdown in GC1-spg cells followed by treatment with etoposide (100 μM) for the indicated time. TUBULIN served as a loading control

To further investigate whether there was a special association of mutual regulation between *Lnc10* and the QKI-5 protein, we initially performed a knockdown or overexpression of QKI-5 in GC1-spg cells. As a result, we found no significant differences in *Lnc10* levels after the knockdown or overexpression of QKI-5 (Fig. 5a, b). Previous reports indicated that QKI was involved in the stability of some targeting mRNAs. Given this notion, we employed qRT-PCR to detect the stability of *Lnc10* transcripts after the knockdown of QKI-5 following by treatment with actinomycin D to block new RNA synthesis. However, we observed that the knockdown of QKI-5 had no significant effects on the half-time of *Lnc10* (Fig. 5c). Additionally, Western blotting showed that the knockdown of *Lnc10* following treatment with Cycloheximide (CHX) to block protein translation had no influence on the expression level of QKI-5 (Fig. 5d). These results implied that *Lnc10* represented a “bona fide” binding target for QKI-5 protein.

LncRNAs have been found to exert their regulatory roles by acting competitively with functional proteins. Considering this action, we hypothesized that *Lnc10* may modulate the p38 MAPK signaling pathway via competitively binding to QKI-5. To test this possibility, we examined the effect of *Lnc10* overexpression on the activation of p38 MAPK and apoptosis (Fig. 5e). Western blotting showed that the expression level of cleaved caspase-3 and p38 phosphorylation in *Lnc10*-overexpressing cells (pcDNA3.1-*Lnc10*) were significantly lower than those in the normal control (pcDNA3.1) during 3–9 h; this indicates that the overexpression of *Lnc10* inhibited the p38 MAPK and apoptosis, which was consistent with the loss of function roles of QKI-5 (Fig. 5f). We next determined whether the simultaneous knockdown of QKI-5 was able to rescue the apoptotic phenotype and activation of p38 MAPK induced by the loss of *Lnc10*. Notably, we observed that the expression level of cleaved caspase-3 and p38 phosphorylation in both *Lnc10* and QKI-5 knockdown cells was substantially lower than those in the normal control; this suggests that the simultaneous knockdown of QKI-5 at least in part revert the effect of ASO-*Lnc10* (Fig. 5g). Taken together, these findings demonstrate that *Lnc10* inhibits the p38 MAPK signaling pathway and apoptosis via binding to QKI-5.

### Depletion of *Lnc10* causes spermatogenesis abnormalities by promoting germ cell apoptosis in vivo

**Fig. 6:**
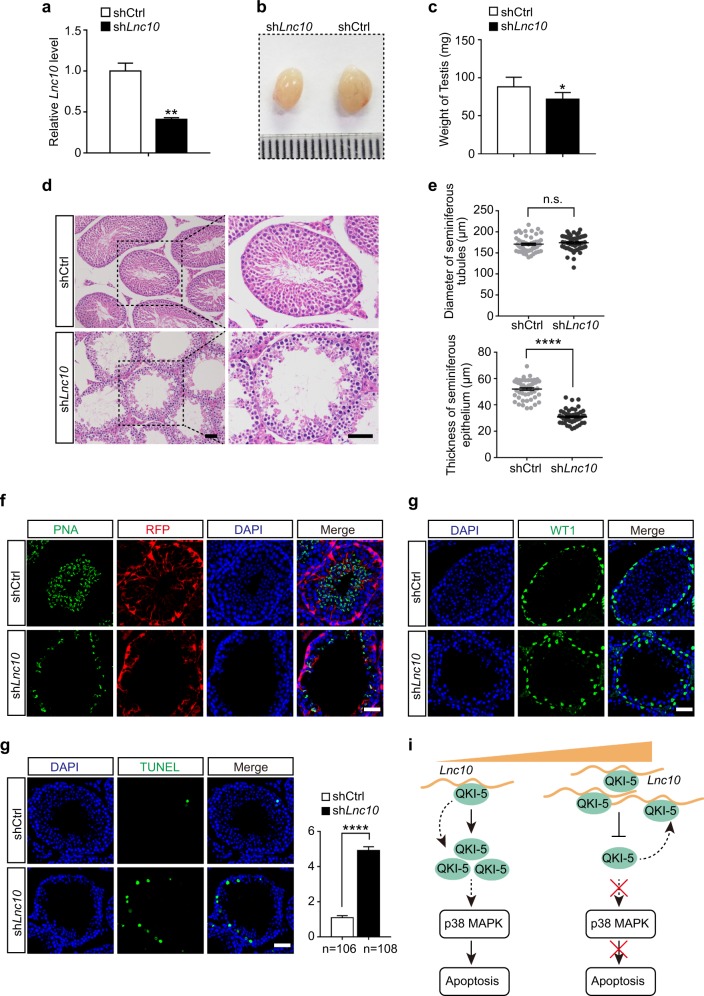
Depletion of *Lnc10* causes spermatogenesis abnormalities by promoting germ cell apoptosis in vivo. **a** qRT-PCR analysis of the shRNA-mediated *Lnc10* knockdown by AAV9. The data represent the mean ± SEM. (**) *p* < 0.01, *t*-test. **b**, **c** Testis morphology and the average weight from the shCtrl and sh*Lnc10* mice. The data represent the mean ± SEM (*n* = 5). (*) *p* < 0.05, *t*-test. **d** H&E staining of testis sections from the shCtrl and sh*Lnc10* mice. Scale bar, 50 μm. **e** Statistical plots of the diameter of the seminiferous tubules (Top) and the thickness of the seminiferous epithelium (bottom) from the shCtrl and sh*Lnc10* mice. The data represent the mean ± SEM of at least 50 seminiferous tubules from 3 mice. (****) *p* < 0.0001, (n.s.) *p* > 0.05, *t*-test. **f** Immunostaining of testis cryosections from the shCtrl and sh*Lnc10* mice for PNA (green) and RFP (red). The nuclei were stained with DAPI (blue). Scale bar, 50 μm. **g** Immunostaining of testis sections from the shCtrl and sh*Lnc10* mice for WT1 (green). The nuclei were stained with DAPI (blue). Scale bar, 50 μm. **h** TUNEL assay on testis sections from the shCtrl and sh*Lnc10* mice. Left: representative staining images; Right: quantification of apoptotic cells in seminiferous tubules. The data represent the mean ± SEM of at least 100 seminiferous tubules from 3 mice. (****) *p* < 0.0001, *t*-test. Scale bar, 50 μm. **i** Proposed working model for *Lnc10* inhibiting QKI-5 downstream p38 MAPK signaling pathway and apoptosis by binding to QKI-5

To further functionally characterize *Lnc10* in mouse spermatogenesis in vivo, we performed a small hairpin RNA (shRNA)-mediated knockdown by adeno-associated virus (AAV9) via a seminiferous tubule microinjection. A qRT-PCR analysis showed that the expression of *Lnc10* was reduced by at least 50% at 4 weeks after the microinjection with AAV9-sh*Lnc10*-RFP (Fig. 6a). In addition, we found that the average weight of *Lnc10*-depleted testes was ~20% lower than that of shCtrl testes (Fig. 6b, c). To further characterize the phenotypes of *Lnc10*-depleted testes, we next examined the testis tissue morphology using H&E stained sections. The results showed that the *Lnc10*-depleted testes exhibited severe morphological defects and a dramatic cell loss in the seminiferous tubules (Fig. 6d). Notably, the thickness of the seminiferous epithelium was substantially reduced by ~50%, but there was no significant change in the diameter of the seminiferous tubules compared with that of the shCtrl testis (Fig. 6e). Immunostaining for PNA, an acrosomal marker, also revealed a deficiency of germ cells in *Lnc10*-depleted testis (Fig. 6f).

As Sertoli cells were also infected by AAV9, we examined the immunostaining for Wilms Tumor 1 (WT1), a Sertoli cell marker, but detected no significant differences in terms of the localization and number of WT1^+^ cells between the shCtrl and *Lnc10*-depleted testes; this suggests that AAV9 infection had no influence on the maintenance of Sertoli cells (Fig. 6g). Given the functional roles of *Lnc10* in apoptosis in vitro, a TUNEL assay was then carried out to evaluate the effect of *Lnc10* depletion on germ cell apoptosis in vivo. Notably, we observed a significant increase in the number of apoptotic germ cells in *Lnc10*-depleted testes. Based on the morphology and position in the seminiferous epithelium, the TUNEL-positive cells were mainly in the spermatogonia and spermatocytes, but few Sertoli cells were TUNEL-positive (Fig. 6h). Collectively, these in vivo functional data reveal that *Lnc10* depletion leads to spermatogenesis defects, which are at least partially attributed to the promotion of apoptosis in germ cells.

## Discussion

Accumulating evidence has documented that QKI, which is extremely conserved from *Drosophila* to mammals, has diverse roles during physiological and pathological processes^32^. However, the precise regulatory roles of QKI in male germline development remain largely elusive. It has been reported that QKI-5 can selectively interact with multiple RNA species, such as pre-mRNA, microRNA, and circRNA, by binding to the QREs located in the target RNAs. Given this model, it is of great interest for us to ask whether there is a class of lncRNAs, including QRE, which can physiologically interact with QKI-5 and plays an important role in spermatogenesis. Through the integrated analysis of RIP-microarray and biological verification, we found that some lncRNAs contained one or more QREs, which were located in their primary RNA sequences. There were also some lncRNAs without a QRE, and these lncRNAs may not directly bind to QKI-5, but indirectly. To some extent, these observations illuminate the binding relationships between QKI-5 and lncRNAs.

A widely proposed hypothesis is that lncRNAs may exert their functions through specific interactions with functional proteins, such as *LncmyoD*^33^, *LncTCF7*^34^, and *Lnc-DC*^35^. Given the lncRNA-protein interactions in association with protein functions, it is tempting to speculate that *Lnc10* binding to QKI-5 may have an effect on the regulatory function of QKI-5. Of interest, we found that *Lnc10* depletion promoted germ cell apoptosis via the activation of p38 MAPK, whereas the simultaneous knockdown of QKI-5 in part revert the effect induced by the loss of *Lnc10*. Indeed, lncRNAs that inhibit protein regulatory functions through competitive binding have been reported, such as Malat1^36^, Gomafu^18^, and lnc-Lsm3b^19^. It is an attractive possibility that *Lnc10* inhibits the regulatory function of QKI-5 through competitive binding, although further investigations are required to determine the precise mechanism. These observations of the *Lnc10*-QKI-5-p38 MAPK regulatory axis may broaden the known regulatory roles of QKI-5. Since QKI-5 is widely expressed in multiple tissues, involved in organ development, and the lncRNA-QKI-5 interaction is associated with the regulatory function of QKI-5, it will be of great interest to identify QKI-5-binding lncRNAs in other systems.

In fact, a number of lncRNAs have been systematically identified in the testes of multiple species. A recent study has revealed critical functions of testis-specific lncRNAs in late *Drosophila* spermatogenesis^37^. Additionally, the knockout of an ultraconserved lncRNA, *THOR*, produces fertilization defects in zebrafish^38^. These findings indicate that lncRNAs may play similar functional roles in male germline development across multiple animal species. However, most lncRNAs have not been functionally characterized in vivo in mouse models. Thus, we performed a shRNA-mediated knockdown by AAV9 to investigate the functional role of *Lnc10* in vivo^39^. Our study demonstrated that the depletion of *Lnc10* caused spermatogenesis abnormalities by promoting germ cell apoptosis, which was consistent with the regulatory effects in vitro.

In summary, this study determines the regulatory role of QKI-5 in male germline development which is involved in the p38 MAPK signaling pathway and germ cell apoptosis. Then, we reveal that the *Lnc10*-QKI-5 interaction is associated with the regulatory function of QKI-5, which inhibits the downstream p38 MAPK pathway and germ cell apoptosis (Fig. 6i). This in vivo functional study indicates that *Lnc10* causes spermatogenesis abnormalities by promoting germ cell apoptosis. Thus, our study provides a potential strategy to characterize the biological significance of the lncRNA-RBP interaction in male germline development.

## Materials and methods

### Cell culture and reagents

The distinct germ cell types were isolated from the testes of ICR mice. GC1-spg cell lines were purchased from the American Type Culture Collection (ATCC, Manassas, USA) and cultured in Dulbecco’s modified Eagle’s medium (Gibco, NY, USA) supplemented with 10% fetal bovine serum (FBS; Gibco, NY, USA) and 1% penicillin-streptomycin (Invitrogen, CA, USA) at 37 °C with 5% CO_2_. For cell treatment, 100 μM Etoposide (Selleckchem, Houston, TX, USA) was incubated with cells for the periods of time. Actinomycin D (5 μg/mL) was purchased from AMRESCO. Cycloheximide (CHX, 100 μg/mL) was purchased from Sigma-Aldrich (St. Louis, MO, USA). SB203580 (10 μM) was purchased from Selleck Chemicals (Houston, TX, USA).

### Plasmid constructs

The cDNA of QKI-5 was PCR-amplified and subcloned into p3XFLAG-CMV™-14 EXPRESSION VECTOR using ClonExpress II One Step Cloning Kit (Vazyme Biotech, Nanjing, China), named Flag-QKI-5. The cDNA of *Lnc10* was PCR-amplified and subcloned into pcDNA3.1 (+) vector using ClonExpress II One Step Cloning Kit (Vazyme Biotech), named pcDNA3.1-*Lnc10*.

### Transient transfection

Plasmids were transiently transfected into GC1-spg cells with Lipofectamine 3000 Transfection Reagent (Invitrogen) according to the manufacturer’s instructions. The siRNA specific to murine QKI-5 (forward: GAACAGAGCAGAAAUCAAAtt; reversed: UUUGAUUUCUGCUCUGUUCaa) and silencer negative control siRNA were transfected into GC1-spg cells using Lipofectamine RNAiMAX Transfection Reagent (Invitrogen). LncRNA Smart Silencer (RiboBio, Guangzhou, China) specific to murine *Lnc10* and Smart Silencer control were transfected into GC1-spg cells using Lipofectamine RNAiMAX Transfection Reagent (Invitrogen).

### Western blotting and antibodies

Total cell and tissues lysates were prepared in 1 × sodium dodecyl sulfate buffer. Cell or Tissue lysates were subjected to SDS/PAGE and transferred onto a PVDF membrane. The protein levels were quantified by densitometry using AlphaEaseFC software. The following antibodies were used for Western blotting analysis. The anti-QKI-5 (#AB9904) was purchases from Millipore Company (MA, USA). The anti-JNK (#9252), phospho-JNK (#9255), ERK1/2 (#9102), phospho-ERK1/2 (#9101), P38 (#9212), phospho-P38 (#9211), cleaved caspase-3 (#9664), cleaved PARP (#9548), TUBULIN (#2128) were purchases from Cell Signaling Technology (Danvers, MA, USA).

### Immunostaining

The testes dissected from mice was fixed in 4% paraformaldehyde for 24 h and then incubated in sucrose-PBS solution (10% sucrose for 1 h, 20% sucrose for 1 h, and 30% sucrose overnight) at 4 °C. Frozen sections were cut to a thickness of 7 μm using the Leica CM1950 (Leica, Solms, Germany). Cryosections were washed in PBS and permeabilized with PBS containing 0.5% Triton X-100. The slides were blocked with 5% BSA and then incubated with primary antibody at 4 °C overnight. AlexaFluor™ conjugated secondary antibody was added for 1 h at room temperature. Finally, the slides were mounted with SlowFade™ Gold Antifade Mountant with DAPI (Invitrogen) and observed under LSM 780 confocal microscope (Zeiss, Germany) for fluorescent signal analysis.

### Total RNA isolation and quantitative real-time PCR

Total RNA was extracted from cell or tisue using Trizol reagent according to manufacturer’s instructions and quantified using the NanoDrop 2000 spectrophotometer (Thermo Scientific). Total RNA (1 μg) was reverse-transcribed to cDNA using RevertAid First Strand cDNA Synthesis Kit (Thermo Scientific). Quantitative real-time PCR was performed in Step One ABI real-time PCR System through PowerUp SYBR Green Master Mix (Applied Biosystems, Foster City, CA, USA). The primers used for qRT-PCR were listed in Supplemental Table S1.

### RNA immunoprecipitation (RIP)

The testes were homogenized in ice-cold PBS using a homogenizer and were resuspended in 800 μl lysis buffer (10 mM Tris, pH 7.5, 100 mM NaCl, 2.5 mM MgCl_2_, 0.05% NP-40, 1.5 mM DTT, 1× Protease Inhibitor Cocktail and 200 U/ml RNase Inhibitor). After the lysates were centrifuged at 12,000 rpm for 15 min, the supernatants were precleared with protein A beads (Roche, Mannheim, Germany) in lysis buffer. Then, the preclear lysates were used for RIP with anti-QKI-5 and rabbit isotype control IgG antibodies. RNA-IP was carried out for 4 h at 4 °C. The beads were washed four times with wash buffer, followed by extraction with proteinase K (20 mg/μl) master mix (2.5 μl proteinase K, 0.5 μl 20% SDS, 100 μl wash buffer) at 55 °C for 15 min. The RNA for each RNA-IP sample was extracted with acid phenol/chloroform and was treated with DNase I. The RNA sample was used for a lncRNA microarray assay or a reverse transcription assay.

### Agilent mouse lncRNA Microarray assay

The RNA for the RNA-IP sample was quantified by the NanoDrop ND-2000 (Thermo Scientific), and the RNA integrity was assessed using an Agilent Bioanalyzer 2100 (Agilent Technologies). Sample labeling, microarray hybridization and washing were performed based on the manufacturer’s standard protocols, provided by the Shanghai Oebiotech Co Ltd (Shanghai, China). Briefly, the total RNA was transcribed to double-stranded cDNA, synthesized into cRNA and labeled with Cyanine-3-CTP. The labeled cRNAs were hybridized onto the microarray. After washing, the arrays were scanned by the Agilent Scanner G2505C (Agilent Technologies). Feature Extraction software (version 10.7.1.1, Agilent Technologies) was used to analyze the array images to obtain the raw data. GeneSpring was utilized to finish the basic analysis with the raw data. The raw data were normalized with the quantile algorithm. Differentially expressed mRNAs or lncRNAs were then identified by their fold change. The threshold that was set for the up-regulated genes was a fold change >=2.0. The QKI-5 binding mRNAs were listed in Supplemental Table S3. The QKI-5 binding lncRNAs were listed in Supplemental Table S4;

### RNA pull-down Assay

For RNA pull-down assay in GC1-spg cells, we designed 8 tiled antisense probes covering the sequence of *Lnc10* using an online design tool (http://www.singlemoleculefish.com/). Probes were synthesized and labeled with Biotin at 3′ end (Invitrogen). RNA pull-down assay was performed according to a previously published protocol^40^. The probe sequences used for RNA pull-down in GC1-spg cells are listed in Supplemental Table S2.

For RNA pull-down assay in isolated type B spermatogonia and pachytene spermatocyte, we used the Pierce™ Magnetic RNA-Protein Pull-Down Kit (Thermo Scientific) according to manufacturer’s instructions.

### Cell apoptosis assay

For GC1-Spg cell apoptosis detection, FITC Annexin V Apoptosis Detection Kit I (BD Pharmingen, San Diego, CA, USA) was used to stain cells according to manufacturer’s instructions. Apoptotic cells were determined by FACS analysis (BD Pharmingen).

For cell apoptosis in the testicular sections, apoptosis was evaluated via a terminal deoxynucleotidyl transferase-mediated dUTP nick end labeling (TUNEL) assay using In Situ Cell Death Detection Kit (Roche) following the manufacturer’s instructions.

### Isolation of Nuclear and Cytoplasmic RNAs

Germ cells (1 × 10^6^) were collected from the testis. Nuclear and cytoplasmic RNA fractions were isolated from Germ cells using the PARIS isolation kit (Thermo Scientific) according to the manufacturer’s instructions. Small nuclear RNA U6 and *Actb* were used as positive controls for nuclear and cytoplasmic RNAs, respectively.

### Isolation of Germ cells, Sertoli cells and Leydig cells

The isolation of germ cells, Sertoli cells and Leydig cells was performed according to a previously described protocol^41,42^.

### Microinjection of virus particles

Virus particles were introduced into the seminiferous tubules via the efferent duct in 3-week-old ICR male mice. Approximately 15 μL of virus particles containing 0.04% Trypan blue solution (Sigma-Aldrich) were injected into the seminiferous tubules using a glass microcapillary pipette with a tip diameter of 40 μm. One testis was injected with AAV9-shCtrl-RFP; the contralateral testis was injected with AAV9-sh*Lnc10*-RFP. All animal experiments were performed with the approval of the Research Ethics Committee of Peking Union Medical College.

### Statistical analysis

All experiments were performed at least in triplicate and the values were presented as means ± SEM. Student’s *t*-test (two-tailed) was performed to analyze the data. *P*-value of <0.05 was considered to be statistically significant.

## Supplementary information

Table S1

Table S2

Table S3

Table S4

Supplementary figure legends

Supplementary Figure 1

Supplementary Figure 2

Supplementary Figure 3
