# Supplementary material for: A long noncoding RNA binding to QKI-5 regulates germ cell apoptosis via p38 MAPK signaling pathway
Source: Cell Death Dis. 2019 Sep 20;10(10):699. doi: 10.1038/s41419-019-1941-2 (PMC6754436; doi:10.1038/s41419-019-1941-2)
Supplement: Supplementary file 5 — Supplementary figure legends [file 41419_2019_1941_MOESM5_ESM.docx]

**Supplemental Figure S1:**  (a) Gene Ontology (GO) term enrichment analysis of the QKI-5-binding mRNAs as determined by an RIP-microarray in adult mouse testis. (b) Western blotting analysis of the phosphorylation and total levels of ERK1/2, JNK, and p38 MAPK in siNC and siQKI GC1-spg cells. TUBULIN served as the loading control. The relative protein level was quantified by densitometry on at least three independent experiments. (n.s.) *p*>0.05, (**) *p*<0.01, *t*-test. (c) Western blotting analysis of the phosphorylation and total levels of p38 MAPK in the normal control and QKI-5 overexpression GC1-spg cells. TUBULIN served as the loading control. The relative protein level was quantified by densitometry on at least three independent experiments. (n.s.) *p*>0.05, (*) *p*<0.05, *t*-test.

**Supplemental Figure S2:** (a) RIP-PCR analysis of lncRNA H19, not including a QRE element, in GC1-spg cells. (b) RT-PCR analysis of the subcellular localization of *Lnc10* by the fractionation of GC1-spg cells. U6 RNA served as a positive control for nuclear gene expression. *Gapdh* mRNA served as a positive control for cytoplasmic gene expression. Nuc RNA: Nuclear fraction RNA; Cyt RNA: Cytoplasmic fraction RNA.

**Supplemental Figure S3:** RNA pull-down analysis of the binding of *Lnc10* to QKI-5 in isolated SG-B (a) and pacSC (b) as detected by a Western blotting assay. TUBULIN served as the loading control.
