# Supplementary figures and images for: A long noncoding RNA binding to QKI-5 regulates germ cell apoptosis via p38 MAPK signaling pathway
Source: Cell Death Dis. 2019 Sep 20;10(10):699. doi: 10.1038/s41419-019-1941-2 (PMC6754436; doi:10.1038/s41419-019-1941-2)

Supplemental Figure S1

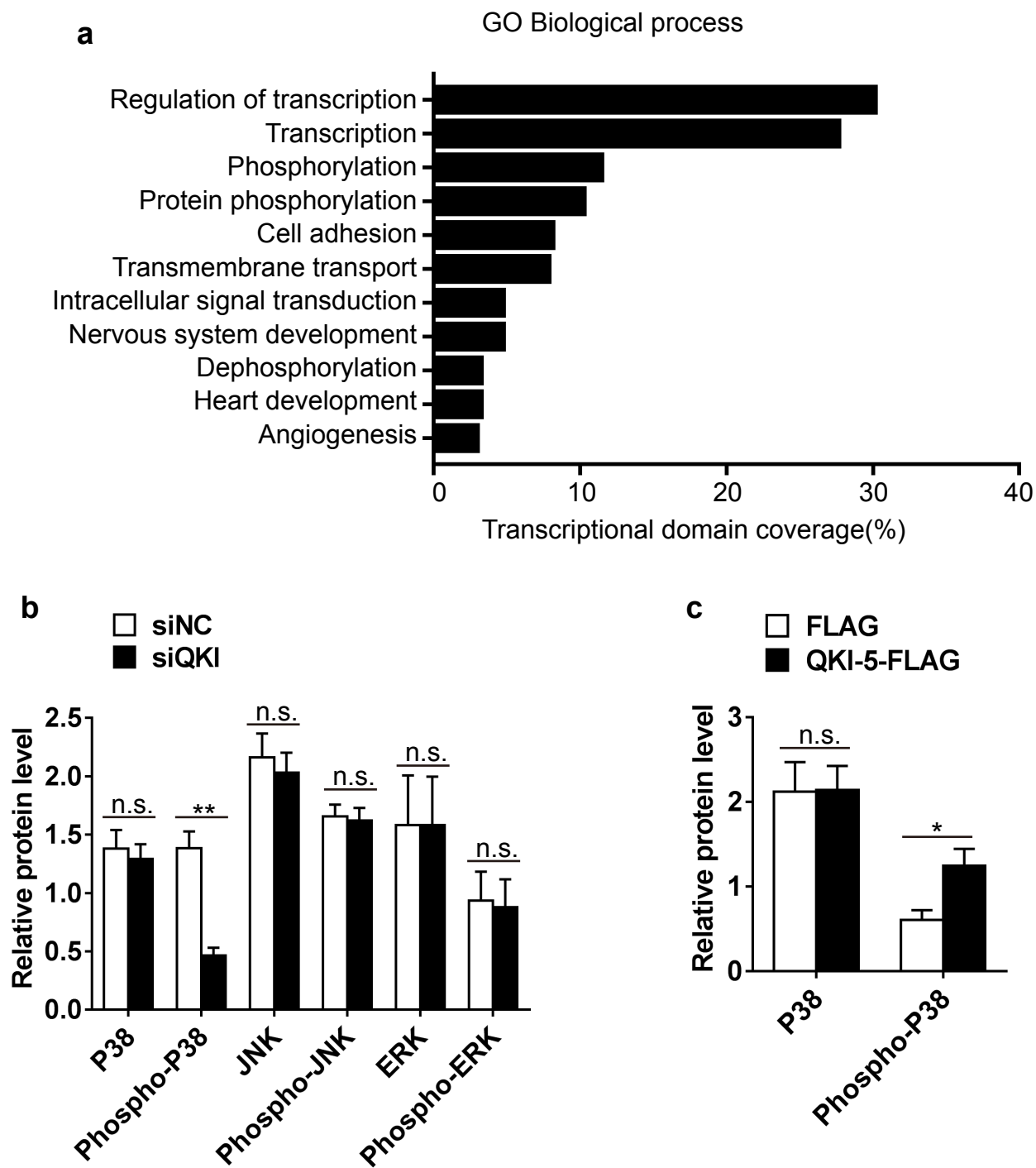

Supplement: Supplementary file 6 — Supplementary Figure 1 [file 41419_2019_1941_MOESM6_ESM.pdf]

Supplemental Figure S2

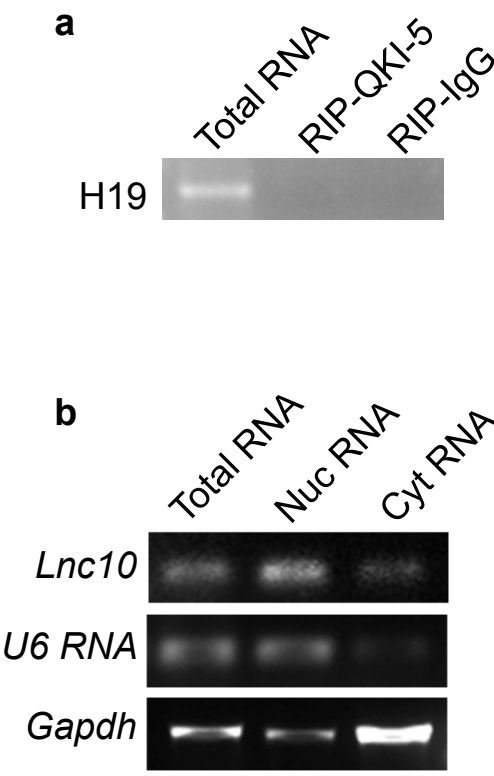

Supplement: Supplementary file 7 — Supplementary Figure 2 [file 41419_2019_1941_MOESM7_ESM.pdf]

Supplemental Figure S3

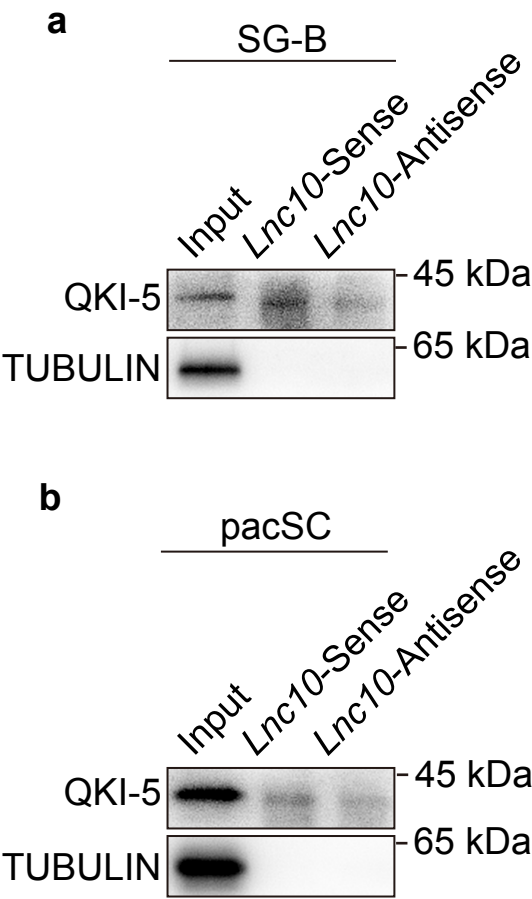

Supplement: Supplementary file 8 — Supplementary Figure 3 [file 41419_2019_1941_MOESM8_ESM.pdf]
